# Theoretical Study of the Defects and Doping in Tuning the Electrocatalytic Activity of Graphene for CO_2_ Reduction

**DOI:** 10.3390/nano13152273

**Published:** 2023-08-07

**Authors:** Xiao Su, Fanqi Meng, Xiang Li, Yueying Liu, Hongwei Tan, Guangju Chen

**Affiliations:** Key Laboratory of Theoretical and Computational Photochemistry, Ministry of Education, College of Chemistry, Beijing Normal University, Beijing 100875, China

**Keywords:** graphene, ECO2RR, first-principle calculation, doping, defect

## Abstract

The application of graphene-based catalysts in the electrocatalytic CO_2_ reduction reaction (ECO_2_RR) for mitigating the greenhouse effect and energy shortage is a growing trend. The unique and extraordinary properties of graphene-based catalysts, such as low cost, high electrical conductivity, structural tunability, and environmental friendliness, have rendered them promising materials in this area. By doping heteroatoms or artificially inducing defects in graphene, its catalytic performance can be effectively improved. In this work, the mechanisms underlying the CO_2_ reduction reaction on 10 graphene-based catalysts were systematically studied. N/B/O-codoped graphene with a single-atom vacancy defect showed the best performance and substantial improvement in catalytic activity compared with pristine graphene. The specific roles of the doped elements, including B, N, and O, as well as the defects, are discussed in detail. By analysing the geometric and electronic structures of the catalysts, we showed how the doped heteroatoms and defects influence the catalytic reaction process and synergistically promoted the catalytic efficiency of graphene.

## 1. Introduction

Extensive use of fossil energy by modern industry exacerbates carbon dioxide (CO_2_) emissions, which have been recognized as the primary driver of climate change. Global energy-related emissions of carbon dioxide hit a record high in 2022. Urgent action is needed to limit the impact of increased coal and oil usage. Since the alternatives to fossil energy are still limited, recycling CO_2_ into valuable chemicals, fuels, and materials has emerged as an opportunity to reduce the emissions of these products [1,2,3]. Biological, thermochemical, photochemical, and electrochemical techniques for CO_2_ reduction are being widely studied. Among various means of CO_2_ conversion, the electrocatalytic CO_2_ reduction reaction (ECO_2_RR) has the ability to convert CO_2_ into usable fuels under mild conditions with controllable reaction rates and product selectivity [4,5,6]. Multiple reduced products, such as CO, methane, methanol, and ethanol, are generated from the ECO_2_RR process.

CO_2_ reduction is a very challenging task due to the chemical inertness of CO_2_ [7]. Hence, efforts have been made to develop catalysts for CO_2_ reduction with high efficiency and selectivity. Numerous heterogeneous and molecular transition metal-based catalyst materials have been studied in recent years [8,9,10]. Some noble metal catalysts, such as Au, Ag, and Pd, are known to be highly preferential towards the ECO_2_RR. For example, Au nanoparticles showed enhanced catalytic activity for converting CO_2_ into CO at ultralow potentials, on which CO production started at a very low potential of −0.15 V vs. reversible hydrogen electrode (RHE), and the highest faradaic efficiency (FE) reached 93% at −0.25 V vs. RHE [11]. As reported by Kenis et al., Ag-coated multiwalled carbon nanotubes yielded unprecedentedly high levels of CO production of up to 350 mA cm^−2^ with high FE and 95% selectivity for the production of CO [12]. Although noble metal catalysts showed very good performance in CO_2_ reduction, their application as industrial electrocatalysts is still impractical due to their high price and scarcity. Hence, the recent trend in the development of the catalyst focuses on material design that reduces the overpotential and enhances CO selectivity with lower material costs. Cheap metal-based catalysts, such as Pb, Sn, Zn, and Bi, require higher overpotentials for catalytic CO_2_ reduction and have low current densities. SnO_2_ nanoparticles (<5 nm) exhibit a high total FE of 97% towards the electrochemical reduction of CO_2_ at −0.95 V vs. RHE [13], although the partial current density for formate production is only 64 mA cm^−2^. Hydrocerussite (Pb_3_(CO_3_)_2_(OH)_2_) can serve as a stable and active phase to produce formate with an optimum FE of 96.4  ±  0.9% under −0.92 V vs. RHE, but it is not environmentally friendly [14]. Cu-based catalysts showed considerable activity for the production of highly reduced C_1_ and C_2+_ products; however, the commercialization of the catalysts is hindered by their low selectivity [15,16].

Nevertheless, metal-free electrocatalysts have recently emerged as candidates for a new generation of catalysts due to their low cost, structural tunability, strong stability and environmental friendliness [17]. Materials including conducting polymers, pyridinium derivatives, aromatic anion radicals, and heteroatom-doped carbon materials were hence substantially investigated for their ECO_2_RR catalysis activities. Carbon-based metal-free electrocatalysts [18] (C-MFECs), such as fullerenes, carbon nanotubes, graphene, and graphdiyne, have attracted extensive attention due to their potential application as catalysts not only for the ECO_2_RR but also for reactions, such as the nitrogen reduction reaction (NRR), oxygen reduction reaction (ORR), and hydrogen peroxide production reaction (H_2_O_2_PR) [19]. In particular, graphene is a new nanomaterial with the highest strength, best electrical conductivity, and excellent carrier mobility found thus far. It has become a natural candidate for use as a catalyst or catalytic support. However, due to its simple composition of carbon, the application of graphene is limited in several aspects, such as the difficulty of activating reactant molecules by neutral carbon atoms in the graphene basal plane, weak adsorption of the reaction intermediates on the graphene surface, and relatively low electroreduction activity of pristine graphene. The introduction of heteroatoms is a practicable way to improve the electrocatalytic performance of graphene. Baek et al. studied the catalytic activity of edge-selectively sulfurized graphene nanoplatelets (SGnP), which demonstrated higher electrocatalytic activity and better fuel selectivity with longer-term stability than pristine graphite and commercial Pt/C electrocatalysts [20]. Theoretical calculations showed that electronic spin density variation caused by sulphur doping played a key role in its high activity. Graphene-based carbon nitride nanosheets showed high performance for oxygen reduction due to the higher electrical conductivity with the introduction of pyridinic N [21]. Two or more different elements codoped in graphene-based materials might also induce synergistic effects to further promote catalytic efficiency. Tuneable N/B-codoped graphene showed excellent oxygen reduction electrocatalytic activity, as reported by Dai et al. [22]. First-principle calculations revealed that the improved catalytic activity of N/B-codoped graphene resulted from its smaller band gap and larger positive spin and charge density polarization. Hu et al. investigated the origin of doping-induced activity variation in graphene by systematically studying the electroreduction reaction of triiodide [23]. Theoretical calculations indicated that nitrogen doping generated a more delocalized electron-donating area on the surface of the catalyst, which enhanced atomic I adsorption, while sulphur doping provided a localized structural distortion, which caused the nearest C to become coordinatively unsaturated sp^3^ hybridization. In another theoretical study, Lee et al. revealed that the synergistic interaction of the NiCo single-atom dimer on the graphene surface caused an upshift in the *d*-band centre of the catalyst, hence facilitating rapid water dissociation and optimal proton adsorption and accelerating the hydrogen evolution reaction kinetics [24].

Zhao et al. recently synthesized a N/B dual-heteroatom-doped three-dimensional hierarchical porous carbon network (NBPC), which had 2–4 nm mesoporous and microporous structures and exhibited 83% CO_2_-to-CO FE at a low overpotential of 290 mV with excellent stability for 20 h [25]. The ECO_2_RR reaction catalysed using the NBPC catalyst exclusively produced CO as the carbon product from CO_2_, with H_2_ being the only byproduct. Normally, the ECO_2_RR activity of B single-doped graphene is very poor. However, the N/B-codoped NBPC catalyst showed enhanced catalytic activity for the *E*CO_2_RR with a markedly higher current density and smaller onset overpotential. Tafel analysis indicated that the N/B-codoped NBPC significantly decreased the reaction barrier in the first reduction process of the ECO_2_RR (CO_2_→COOH), which was the potential determining step of the whole reaction. X-ray photoelectron spectroscopy (XPS*)* demonstrated that pyridinic N was the major N species in the NBPC catalysts. BC_3_ and BCO_2_ were the B species, and no boron nitride was detected in the catalyst. Raman spectra showed that NBPC had a higher value of ID/IG (0.57) than B-doped carbon (BPC) (0.54) and N-doped carbon (NPC) (0.47), which indicated that NBPC was a more inhomogeneous structure with more defects in its lattice attributed to the codoped N and B atoms. Zhao et al. conjectured a possible mechanism underlying the CO_2_RR on NBPC, in which pyridinic N facilitated the first single-electron transfer process, around which the positively charged adjacent carbon atoms were the key active sites for enhancing the adsorption of CO_2_. The strong bonding ability of the B atom with O species would be instrumental in the activation of CO_2_ and the conversion of COOH to CO. This mechanism proposed a possible synergetic effect between the doped N and B atoms in the catalyst. However, XPS experiments also detected BCO_2_ species in the catalyst, and whether the O atom played a role in improving the efficiency of the catalytic CO_2_RR reaction in NBPC remained elusive. Previous studies indicated that O species in graphene-based catalysts were able to enhance their catalytic activity in ORR reactions. The O atoms were able to polarize the surrounding atoms and induce charge delocalization in the catalyst, which increased the adsorption of the ORR species on the surface of the catalyst [26]. In the study of the NRR reaction catalysed using the graphdiyne catalyst, Dai et al. also noticed that O doping enhanced the capture of N_2_ molecules by the catalyst, which resulted from the charge redistribution of the catalyst induced with O doping [27].

In addition to heteroatom doping, lattice defects also have a great impact on the electrocatalytic activity of catalysts [28]. Yao noticed that introducing defects into graphene resulted in a much higher current density and smaller Tafel slope than observed when using pristine graphene in the ORR process [29]. Theoretical investigation indicated that the frontier molecular orbitals of the catalyst, which play critical roles in the catalytic reactions, were mainly contributed by the edge atoms around the defects. Artificial surface defects in the activated carbon (AC) catalyst were also found to be able to increase the acetylene hydrochlorination conversion rate from 20.3% to 61.6% [30]. The catalytic performance of AC was linearly correlated with the number of surface defects. The defective sites in the AC showed better performance than pristine ones not only in binding with the reaction intermediates but also in suppressing the reaction barriers. Heteroatom codoping inevitably caused defects in the graphene catalyst [31]. For instance, due to its different coordination modes, pyridinic N doping resulted in a defect adjacent to it in graphene. However, for the catalytic ECO_2_RR reaction catalysed with graphene, whether and how the defects caused by pyridinic N doping play roles are still intriguing unanswered questions. A detailed mechanistic investigation of the CO_2_ reduction process on N/B-codoped graphene-based catalysis not only reveals how heteroatomic doping changes the surface structure of graphene but also elucidates the respective roles played by the doped atoms and the catalytic synergistic effect between them. Since graphene-based catalysts are promising materials for the ECO_2_RR, some theoretical studies have been carried out to investigate their catalytic mechanism. Previous theoretical studies were mainly based on small cluster models, in which finite-size effects were not considered. Norskov showed that for catalysts with sizes larger than 2.7 nm, the quantum size effect could be disregarded, while the periodicity of the material needed to be considered [32].

In the current study, a series of models of graphene with single N/B/O heteroatom doping and multiple heteroatom codoping was built to investigate the catalytic mechanisms underlying CO_2_ electroreduction with first-principle calculations. The effect of the defects formed by single-atom vacancy and multiatom vacancy were studied as well. The results show that the doped pyridinic N and the single-atom defect promote the adsorption and activation of atomic H and CO_2_ molecules, respectively. Although doping of a single B atom cannot lead to satisfactory catalytic efficiency, the electron-deficient B atom is able to reduce the Δ*G* of the CO_2_→*COOH step by interacting with the reactants CO_2_ and *COOH intermediates, which are captured by a single-atom vacancy defect. The synergistic interaction between the N and B atoms could greatly facilitate the catalytic reactions but lead to difficulties in the desorption of the product CO. Further introducing O atoms into the N/B-codoped catalyst resolves the problem of catalyst poisoning, which is beneficial because the extra electrons carried by O fill the empty level above the Fermi level of the catalyst and weaken the back bonding between CO and the catalyst.

## 2. Methods

### 2.1. Computational Models

**Figure 1:**
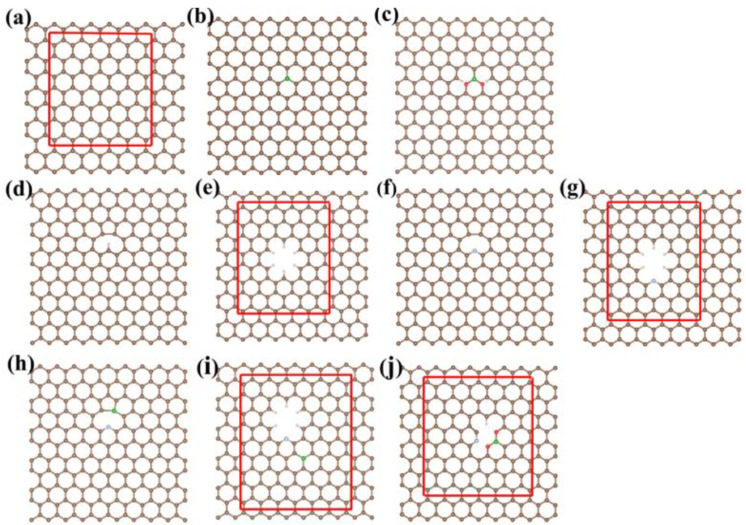
Schematic diagram showing the structure of (**a**) graphene, (**b**) BPC, (**c**) BPC-O, (**d**) void-graphene, (**e**) hole-graphene, (**f**) void-NPC, (**g**) hole-NPC, (**h**) void-NBPC, (**i**) hole-NBPC, and (**j**) void-NBPC-O catalysts. The brown, blue, green, red, and pink spheres represent carbon, nitrogen, boron, oxygen, and hydrogen atoms, respectively.

Because the ECO_2_RR reaction is catalysed on the surface of the porous NBPC catalyst and its pore structure mainly affects the mass transfer process, the three-dimensional hierarchical carbon network of NBPC was reduced to a two-dimensional slab model in our theoretical simulations. The initial periodic pristine graphene system contained a total of 180 carbon atoms and had dimensions of 21.37 Å × 22.20 Å × 18.0 Å (α = β = γ = 90°), corresponding to a 5 × 9 × 1 supercell expanded from the unit cell, with a sufficiently large lateral dimension to accommodate the ECO_2_RR intermediates, as shown in Figure 1a. The vacuum layer in the slab model was set to 18 Å to avoid the interaction between atoms in adjacent unit cells perpendicular to the surface. The catalytic site was set at the centre on the planar surface of the graphene. To better reproduce the catalytic environment, an expanded region with a size of 5 × 6 six-membered rings was relaxed in all the optimization calculations, while the atoms outside the region were fixed to fit the extended surface structure of the catalyst. Based on this pristine graphene model, heteroatom-doped models were further constructed. The B atom was doped in the form of BC_3_ (BPC) and BCO_2_ (BPC-O), as shown in Figure 1b,c. BPC-O was built based on the BPC model, and the two C atoms on the two sides of the B atom were replaced with O atoms to construct the BCO_2_ species. The N-atom was doped in the pyridinic form and set as a single-atom vacancy defect (void-graphene, Figure 1d) and a multiatom vacancy defect (hole-graphene, Figure 1e), including void-NPC (Figure 1f) and hole-NPC (Figure 1g), respectively. The edge atoms around the defects were saturated with H atoms. The models of N/B-codoping graphene were built based on the N-doped models mentioned above. The B atom was doped in the 4 Å range around the N atom. There were seven and five possible B doping sites for void-NBPC and hole-NBPC, respectively, as shown in Figure S1, which were further screened by their relative stabilities. Figure 1h,i displays the most stable models of void-NBPC and hole-NBPC, respectively. The void-NBPC catalyst was selected for further O doping to obtain the three-atom doped model of void-NBPC-O, as shown in Figure 1j. Since the sizes of biatomic and triatomic doping sites and the vacancy defect were beyond the single atom site as in pristine graphene, the relaxation regions in these models were correspondingly extended to ensure that the distance between the dopant atom and the boundary was greater than 5 Å, as shown in Figure 1e,g,i,j.

### 2.2. Computational Details

All DFT calculations were performed using the Vienna Ab Initio Simulation Package [33,34,35] (VASP, version 6.3.0, Vienna, Austria) with dispersion correction [36,37] (DFT + D3). The core-valence electron interactions were described using the projector augmented wave (PAW) method [38], where the energy cut-off of the plane wave basis set was set to 450 eV. The generalized gradient approximation (GGA) was used with the Perdew-Burke-Ernzerhof (PBE) functional to describe the electron exchange-correlation interaction [39]. The force and energy convergence criteria were set to 0.02 eV Å^−1^ and 10^−5^ eV, respectively. A gamma-centred 2 × 2 × 1 *k*-point grid was set in the Brillouin zone, while the *k*-point grid was set to 4 × 4 × 1 for the density of states (DOS) calculation. To estimate the solvent effect on the electrocatalytic reactions, the implicit solvent model of VASPsol [40,41] was used with the dielectric constant set to 80 to represent the water solvent. The adsorption energy (*E_ad_*) of each intermediate species was calculated using the following equation:

*E_ad_* = *E_total_* − *E_surface_* − *E_int_* (1)

where *E_total_*, *E_surface_*, and *E_int_* are the energies of the complex in the adsorption configuration, the isolated catalyst, and the intermediate species, respectively. The free energy change Δ*G* in the reaction was calculated using Equation (2):

Δ*G* = *E_FS_* − *E_IS_* + Δ*E_ZPE_* − *T*Δ*S* (2)

Δ*G*(*U*) = Δ*G + neU* (3)

where *E_FS_* and *E_IS_* represent the energies of the final state (FS) and initial state (IS) obtained with the DFT calculations, respectively; Δ*E_ZPE_* and Δ*S* represent the differences in zero-point energy and entropy with the reaction, respectively, which were calculated using the VASPKIT tool [42]; and the temperature *T* was set to 298.15 K. The Δ*G* in the electrode reaction at different applied potentials *U* can be obtained using Equation (3), where *n* is the number of proton–electron pairs transferred relative to the reactant and *e* is the elementary (positive) charge. The computational electrode model (CHE) was applied to calculate the free energies of the proton–electron transfer steps [43,44].

## 3. Results and Discussion

### 3.1. Reaction Pathways for ECO_2_RR

The complete reaction mechanisms underlying the ECO_2_RR on the surfaces of 10 graphene-based catalysts were systematically investigated. The adsorption energies (*E_ad_*) of the reactants and reaction products, including CO_2_, H, and CO, on each of the catalysts were calculated, and the results are listed in Table 1. The adsorption energies were corrected for the solvent effect using VASPsol. The reactions of CO_2_ reduction on these graphene catalyst surfaces follow a common three-step process, including two reduction steps and one product release step, as follows: CO_2_ + H^+^ + *e*^−^→*COOH, *COOH + H^+^ + *e*^−^→*CO + H_2_O and *CO→CO. In each of the reduction steps, a H atom is transferred to the substrate CO_2_ through the PCET process, by which the substrate acquires a proton from the adsorbed H and obtains an electron from the catalyst. Because an electron is transferred in each of the two reaction steps, the reaction is influenced by the applied potential. Furthermore, the reaction is also expected to depend on the strength of the proton donor. The stronger the proton donor strength, the more H atoms adsorb on the catalyst surface. The calculated free energy data for the catalytic reactions on different catalysts are tabulated in Table 2. The overpotential at equilibrium potential (the rightmost column in Table 2) was used to evaluate the catalytic activity of these catalysts [43,45].

#### 3.1.1. ECO_2_RR on Pristine Graphene and the Effect of the Defects

The calculated free energy profiles of the catalytic CO_2_ reduction reactions on the surfaces of perfect pristine graphene, void-graphene, and hole-graphene, as well as the corresponding structures of the reaction intermediates, are shown in Figures S2–S4. On the pristine graphene surface, CO_2_ adsorption is very weak, with an adsorption energy of only −0.15 eV. The CO_2_ molecule maintains linear conformation, and the distance measured between the C atom in the CO_2_ molecule and the graphene surface is 3.32 Å (Figure S2a). The Bader charge analysis [46,47,48] indicates that there is barely any charge exchange between the catalyst surface and CO_2_. The CO_2_ molecule remains in a resting state on the perfect graphene catalyst surface. The adsorption of the other reactant, the H atom, is a thermodynamically unfavourable process (*E_ad_* = 1.31 eV, Table 1). Therefore, the CO_2_ reduction reaction on pristine graphene follows the single molecular Eley–Rideal (ER) mechanism. As shown in Figure S2, the free energy changes in the two reduction steps, CO_2_→*COOH and *COOH→*CO, are 2.45 eV and −1.46 eV, respectively (Table 2). Since there are two electrons being transferred in the whole reduction process, and based on the 0.50 eV free energy difference between the initial reactant CO_2_ and the final product CO, the equilibrium potential (*U_eq_*) of the reaction is calculated to be −0.25 V vs. standard hydrogen electrode (SHE). Usually, an operating potential can be applied such that the energetic change demonstrates a definite downhill along the entire reaction pathway. For the ECO_2_RR on pristine graphene, the operating potential is 2.45 eV. Accordingly, a 2.20 eV overpotential is required for the potential determining step CO_2_*→COOH, which is the potential difference between the equilibrium and operating potentials. These results indicate that the ECO_2_RR reaction is not able to occur on the pristine graphene surface at room temperature, which agrees with the experimental results.

Compared to perfect pristine graphene, CO_2_ adsorbs more strongly on the void-graphene surface with an adsorption energy of −0.64 eV. As shown in Figure S3a, the CO_2_ molecule adsorbs on the defect site of the void-graphene and bends out from the linear conformation with the angle ∠C=O=C decreasing to 119.29°. The CO_2_ molecule presents an asymmetrical conformation with the proximal C=O bond elongating to 1.36 Å, while the distal C=O bond remains at a shorter length of 1.21 Å; thus, the CO_2_ molecule is activated due to the strong chemical adsorption with the catalyst. The Bader charge analysis also confirmed the activation of CO_2_, obtaining a 0.64 *e* electronic charge on the CO_2_ molecule calculated from the catalyst. The other reactant, atomic H, competes for the same adsorption site with CO_2_ on the void-graphene surface, which has an adsorption energy of −0.34 eV (Table 1). It adsorbs weaker on the catalyst than CO_2_. Hence, the reduction reaction on the void-graphene surface follows the single molecule ER mechanism. The free energy change (Δ*G*) in the first reduction step (CO_2_→*COOH) on the void-graphene considerably decreases to 0.97 eV. Furthermore, our calculations indicate that the following reaction step *COOH→*CO is able to spontaneously proceed with a Δ*G* of −2.76 eV (Table 2). Accordingly, the overpotential calculated for CO_2_ reduction on void-graphene is 0.72 eV, which is corrected with the equilibrium potential. Evidently, the void defect on graphene is able to activate CO_2_ molecules and remarkably promote the catalytic activity of the catalyst. However, our calculations also indicate that void-graphene has a very strong interaction with the product CO. The desorption energy of CO is as high as 2.29 eV (Table 2, Figure S3c,e); thus, CO desorption becomes the rate-limiting step in the overall ECO_2_RR process. The strong adsorption of CO on the void defect site prevents it from being released and leads to catalyst poisoning at room temperature, which cannot be compensated even under a higher applied potential.

On hole-graphene, the adsorption of CO_2_ is very weak (*E_ad_* = −0.14 eV, Table 1), which is similar to that on perfect pristine graphene. The Bader charge analysis indicates that there is barely any electron transfer from the catalyst to the adsorbed CO_2_ molecule. The net charge on CO_2_ is only −0.01 *e*. CO_2_ maintains a symmetrical linear conformation with one of the O atoms inclined to the hole on the graphene surface (Figure S4a). Although hole-graphene does not enhance the adsorption of CO_2_, it promotes CO_2_ reduction to CO; the Δ*G* values of the two steps that produce *COOH and *CO intermediates decrease to 1.30 eV and −0.45 eV, respectively. Since the catalyst does not show an enhanced interaction with the reactant CO_2_, the lower Δ*G* in the CO_2_→*COOH step is mainly dictated by the stronger binding of the intermediate *COOH on the catalyst surface. The desorption free energy of the product CO is −0.34 eV. Accordingly, the overpotential of CO_2_ reduction on the surface of the hole-graphene surface is reduced to 1.05 eV (Table 2). The catalytic activity of hole-graphene lies between that of pristine graphene and void-graphene.

#### 3.1.2. Monoatomic-Doped Graphene

##### N Doping

**Figure 2:**
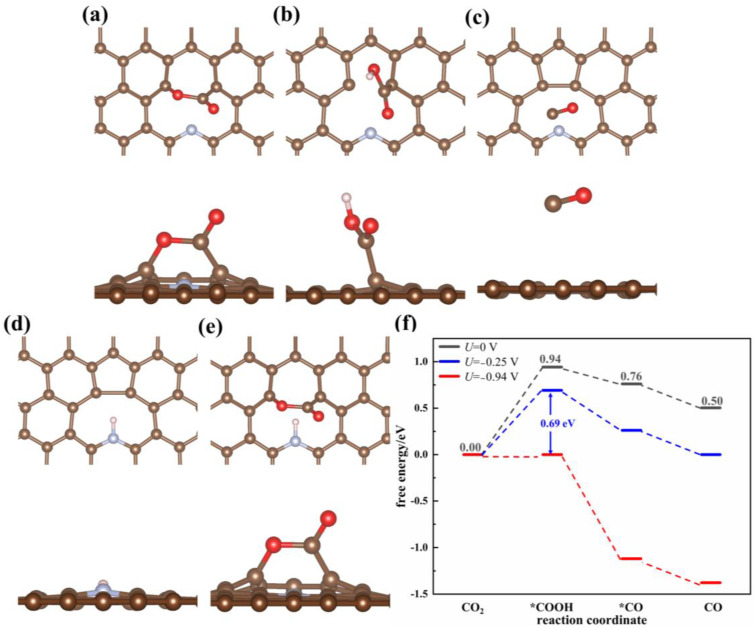
Optimized adsorption configurations of (**a**) CO_2_, (**b**) COOH, (**c**) CO, (**d**) H, and (**e**) CO_2_/H on the void-NPC surface. (**f**) Free energy profile of CO_2_ reduction on the void-NPC surface in the solvation environment at different applied potentials. An asterisk represents a free adsorption site.

N atoms can be doped in graphene in three possible forms: pyridinic N, pyrrolic N and graphitic N. Using experiments, it was demonstrated that pyridinic N is the major N species in N-doped graphene. Pyridinic N doping inevitably introduces defects into graphene. Hence, pyridinic N-doped graphene with single-atom vacancy (void-NPC) and multiatom vacancy defects (hole-NPC) were both investigated. The calculated free energy profiles and optimized reaction intermediates on the surfaces of void-NPC and hole-NPC are shown in Figure 2 and Figure S6, respectively. The adsorption strength of CO_2_ on the void-NPC surface is markedly enhanced compared to pristine graphene (E_ad_ = −0.68 eV). CO_2_ adopts a side-on conformation on the surface of the catalyst, with the O=C=O angle bending to 119.37° (Figure 2a). The proximal C-O bond in the CO_2_ molecule, which lies parallel to the catalyst surface, asymmetrically extends to 1.36 Å, presenting characteristics of a single bond. The other C=O bond is much shorter, with a length of 1.22 Å. The Bader charge analysis indicates that the electron population of −0.64 e is transferred from the catalyst to the CO_2_ molecule. The electron localization function (ELF) also suggests that the electrons are more localized between void-NPC and CO_2_ (Figure S5a) compared with pristine graphene (Figure S5b), and the interaction between them is stronger. The geometric and electronic structures of the adsorbed CO_2_ on the surface of void-NPC, as well as its adsorption energy, are very similar to those on void-graphene; thus, the single carbon atom defect, but not the doped nitrogen atom, plays the critical role in interacting and activating CO_2_. The doped pyridinic N, on the other hand, acts as the site for atomic hydrogen adsorption. As calculated, the adsorption energy of the H atom with pyridinic N in void-NPC is −0.75 eV. The Bader charge analysis indicates that there is a polar single bond formed between pyridinic N and hydrogen, in which the charges on the N and H are −1.31 e and +0.52 e, respectively. Clearly, N doping greatly enhances the adsorption of H on the catalyst surface and facilitates the combination of atomic hydrogen and CO_2_ as a proton in the subsequent reduction reaction. Since CO_2_ and H are both able to adsorb on void graphene at different sites, the CO_2_ reduction reaction follows the bimolecular adsorption Langmiur-Hinshelwood (LH) mechanism. The calculated ΔG values for the two reduction steps, CO_2_→*COOH and *COOH→*CO, are 0.94 eV and −0.18 eV, respectively. After correction using the equilibrium potential of −0.25 V, the thermodynamic energy barrier for the first reduction step to produce the *COOH intermediate is 0.69 eV (Figure 2), which is the potential determining step of the whole catalytic reaction on the void-NPC surface. Accordingly, the operating potential of the void-NPC catalyst is −0.94 V, with which the energy profile demonstrates definite downhill along the overall reaction pathway. Compared to pristine graphene, the overpotential of void-NPC is reduced by 1.51 eV. The product CO is able to spontaneously desorb from the surface of void-NPC with a Δ*G* value of −0.26 eV, which is conducive to renewable catalytic sites. Summarizing the calculation results, single N-doping is able to improve the catalytic activity of the graphene-based catalyst to a higher extent than single B atom doping, which is consistent with the experiments.

Interestingly, in contrast to void-NPC, the adsorption of CO_2_ on the surface of hole-NPC is very weak, which retains a symmetrical linear configuration in the resting state and tilts towards the centre of the hole defect. The adsorption energy of CO_2_ is only −0.19 eV (Figure S6a). The proximal O atom in CO_2_ departs 2.07 Å from the catalyst surface. The net charge on the CO_2_ molecule is only −0.02 *e*. On hole-NPC, atomic H also adsorbs on the pyridinic N with an adsorption energy of −0.42 eV (Table 1). Similarly, the N-H bond is polarized with −1.29 e and +0.48 e charges on the N and H atoms, respectively. Hence, the reduction reaction follows the bimolecular LH mechanism to produce the intermediate *COOH, (Figure S6e), with which the free energy increases by 1.30 eV. Evidently, hole-NPC does not promote the efficiency of the first CO_2_ reduction step as significantly as void-NPC. The free energy change in the second reaction step, within which *COOH cleaves to *CO and *OH, is −0.59 eV. Desorption of the product CO from hole-NPC is spontaneous with a Δ*G* of −0.21 eV. The overpotential of the whole reaction is 1.05 eV. The operating potential of the whole reaction is −1.30 V; thus, the reaction on the surface of hole-NPC is able to spontaneously proceed.

##### B-Doping

**Figure 3:**
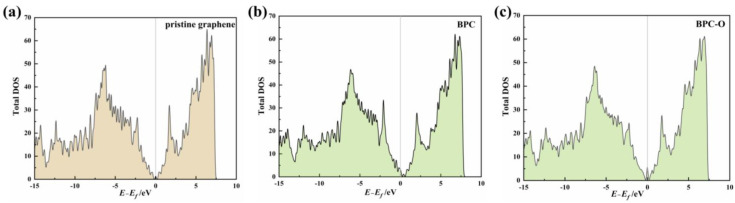
Total DOS of (**a**) pristine graphene, (**b**) BPC, and (**c**) BPC-O catalysts.

For single-B-atom-doped graphene (BPC), the calculated free energy data for the ECO_2_RR process are listed in Table 1. The optimized configurations of the reaction intermediates are presented in Figure S7. The structure of the reactant indicates that BPC does not activate the adsorbed CO_2_, which maintains a symmetrical linear configuration as in the resting state. The C atom in CO_2_ departs 3.10 Å from the catalyst surface (Figure S7a). The charge carried by CO_2_ is only −0.02 e. Its adsorption energy is only −0.15 eV. The adsorption of the other reactant, atomic H, is unfavourable, with an adsorption energy of 0.56 eV. Hence, the adsorption of the reactants on BPC is very similar to that on pristine graphene. The catalytic reaction follows the ER mechanism. The CO_2_→*COOH step is the potential determining step with a ΔG value of 1.52 eV, while the subsequent *CO production step and its desorption are both spontaneous. The overpotential of CO_2_ reduction on BPC is 1.27 eV after correction with the equilibrium potential *U_eq_* = −0.25 V. Although the overpotential is 0.93 eV lower than that on perfect pristine graphene, the reaction still could hardly proceed at room temperature. The density of states (DOS) analysis indicates that B doping only induces a very slight electron population increase at the Fermi energy level of the catalyst (Figure 3a,b). Since the activity of the catalyst is related to its ability to donate electrons in the reduction reactions, the experimental observation is that BPC is not able to achieve satisfactory catalytic activity even though it has a very small enhancement in the catalytic ability compared to pristine graphene is rationalized.

#### 3.1.3. Diatomic-Doped Graphene

Compared to single-element doping, dual-element doping is able to promote the catalytic efficiency of graphene to a higher extent because it is able to exploit the synergistic effect between the doped heteroatoms. Dual element doping also provides a basis for creating more kinds of lattice defects, vacancies, and active sites for catalysis. Since N/B-codoping and O/B-codoping are two kinds of dual-element doping in graphene that were detected with the experiments, their ECO_2_RR mechanisms were investigated.

O/B-codoped graphene (BPC-O) was built based on the single B-doped graphene model (BPC). The doped B and O atoms form a BCO_2_ species, as detected in the experiment. The free energy profiles of CO_2_ reduction reactions on the BPC-O surface at different applied potentials and the corresponding geometric structures of the reaction intermediates are summarized in Figure S8. Compared to pristine graphene and BPC, BPC-O does not show enhanced adsorption of CO_2_ (E_ad_ = −0.17 eV). Due to its strong interaction with the doped O atom, the adsorption energy of the H atom remarkably decreases to −0.46 eV (Table 1, Figure S8d). Therefore, the first reduction step of CO_2_ follows the LH mechanism. The thermodynamics of the whole ECO_2_RR process are very similar to those of BPC. The overpotential of the ECO_2_RR on BPC-O is 1.28 eV, meaning that there is barely any synergistic effect between the doped O and B atoms. The total DOS of the catalyst also remains unchanged after O/B-codoping when compared to BPC (Figure 3c).

**Figure 4:**
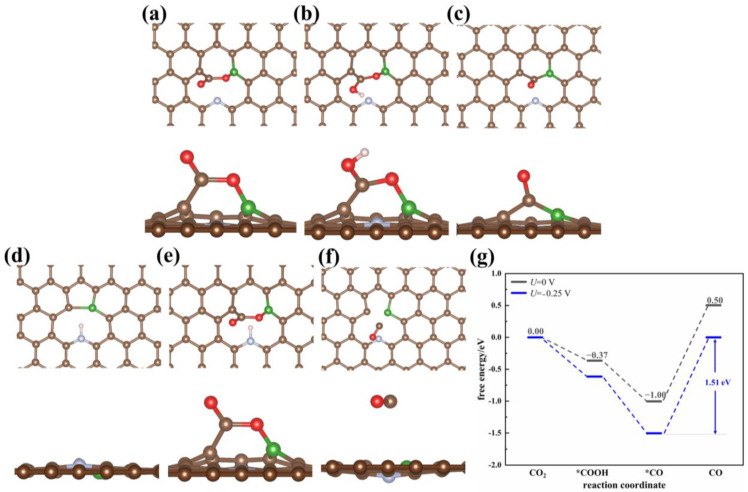
Optimized adsorption configurations of (**a**) CO_2_, (**b**) COOH, (**c**) adsorption state CO, (**d**) H, (**e**) CO_2_/H, and (**f**) dissociative state CO on the void-NBPC surface. (**g**) Free energy profile of CO_2_ reduction on the void-NBPC surface in the solvation environment at different applied potentials. The brown, green, blue, red, and pink spheres represent carbon, boron, nitrogen, oxygen, and hydrogen atoms, respectively. An asterisk represents a free adsorption site.

In contrast to O/B-codoping, N/B-codoping remarkably promotes the catalytic efficiency of the catalyst when compared with single atom-doped BPC and void-NPC. The model of N/B-codoped graphene (void-NBPC) was built by introducing a B atom into void-NPC. The adsorption energy of the CO_2_ molecule to the catalyst void-NBPC is extremely reduced to −1.48 eV. CO_2_ adopts a side-on conformation on the catalyst surface and obtains 0.83 electronic charges from the catalyst, which induces an evident structural variation in CO_2_. The O=C=O bond angle bends to 121.75°. The C-O bond, which lies parallel to the catalyst, converts to a single bond and elongates to 1.34 Å, in which the carbon atom and the oxygen atom form a C-C bond and a B-O bond with the catalyst with bond lengths of 1.50 Å and 1.41 Å, respectively (Figure 4a). Clearly, CO_2_ is highly activated by void-NBPC. The free energy change in the reaction step that reduces CO_2_ to *COOH is −0.37 eV. Among the 10 catalysts studied in this work, void-NBPC is the only one that is able to promote the first reduction step to a spontaneous process. The subsequent reaction step to generate *CO from *COOH is also a spontaneous process with a ΔG value of −0.64 eV. Accordingly, the overpotential for the catalytic reaction is 0 eV at the equilibrium potential. Although the catalytic reactions are efficiently promoted with void-NBPC, our calculations indicate that the reduction product CO has a very strong interaction with the catalyst (E_ad_ = −2.16 eV, Table 1). After correction with the zero-point energy and entropy, the desorption free energy of CO is still as high as 1.51 eV, which becomes the rate-limiting step in the whole catalytic cycle (Figure 4f). The adsorbed CO either leads to poisoning of the catalyst or is further reduced with the catalyst to generate side products (Figure 4c); this result practically excludes void-NBPC as an efficient catalyst to selectively reduce CO_2_ to CO.

We further investigated the N/B-codoped catalyst with a multiatom vacancy defect (hole-NBPC), for which five possible models were built and investigated. Figure 1g shows the sample with the lowest energy and the narrowest energy gap. In this hole-NBPC model, the doped N atom is separated from the B atom by two carbon atoms; hence, it can be speculated that the synergistic effect between them is weak. The free energy profile of the CO_2_ reduction reaction and the optimized intermediate structures on this hole-NBPC surface are shown in Figure S9. The adsorption strengths of the reactants, intermediates, and products on hole-NBPC are very similar to those on hole-NPC. The potential determining step in the reaction still lies in the step CO_2_→*COOH, which can be converted into an overpotential of 0.99 eV after correction with the equilibrium potential of −0.25 V. This overpotential is only 0.06 eV lower than that of hole-NPC, confirming that the doped N plays a critical role in the activity of hole-NBPC and that there is no catalytic synergy effect between N and B.

#### 3.1.4. Triatomic-Doped Graphene

**Figure 5:**
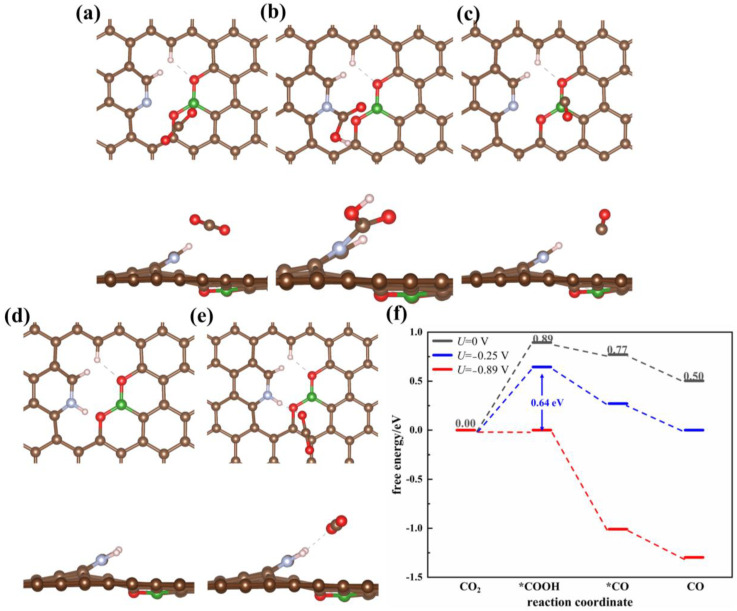
Optimized adsorption configurations of (**a**) CO_2_, (**b**) COOH, (**c**) CO, (**d**) H, and (**e**) CO_2_/H on the void-NBPC-O surface. (**f**) Free energy profile of CO_2_ reduction on the void-NBPC-O surface at different applied potentials. The brown, green, blue, red, and pink spheres represent carbon, boron, nitrogen, oxygen, and hydrogen atoms, respectively. An asterisk represents a free adsorption site.

Although the diatomic doped void-NBPC catalyst presents the best catalytic performance thus far, it suffers from CO poisoning. Notably, catalyst poisoning by CO was not observed in the experiments, which indicated that the efficient catalyst should exist in another form. Hence, we further investigated graphene doped with three heteroatoms, N, B and O, to determine whether it had better catalytic ability and could avoid CO poisoning. A N/B/O triatomic doping model (void-NBPC-O) was built based on void-NBPC. In the void-NBPC-O catalyst, two oxygen atoms reside on the two sides of B to form a BCO_2_ moiety, while N is located on the same edge of the void defect and next to the BCO_2_ moiety (Figure 1j). The optimized reaction intermediates and free energy profile of the CO_2_ reduction reaction on the surface of void-NBPC-O are shown in Figure 5. The CO_2_ molecule is adsorbed above the BCO_2_ moiety with an adsorption energy of −0.19 eV (Figure 5a). CO_2_ tilts towards the B atom in the catalyst due to electrostatic interactions. On the surface of void-NBPC-O, atomic H adsorbs on the pyridinic N with an adsorption energy of −0.59 eV, which departs 2.09 Å away from the O terminal of the reactant CO_2_ molecule. The free energy change in the CO_2_→*COOH reaction is 0.89 eV. For the second reaction step, *COOH→*CO, and desorption of the product CO, the free energy changes are −0.12 eV and −0.27 eV, respectively. Accordingly, the overpotential of the whole catalytic reaction is 0.64 eV after correction with the equilibrium potential of *U_eq_* = −0.25 V. At operating potential U = −0.89 V, the free energy changes are definitely downhill along the entire reaction pathway. This result indicates that void-NBPC-O has the best performance on the whole catalytic reaction among all the catalysts studied in this work and circumvents CO poisoning.

### 3.2. Discussion

As shown by the calculation results, the defects, the doped heteroatoms, and the synergistic effect between them are all able to promote the catalytic performance of graphene-based catalysts to different extents. Herein, the roles of the defects, the doped heteroatoms, and the synergistic effect are discussed based on the detailed geometric and electronic structure analyses.

#### 3.2.1. Defect Effect

**Figure 6:**
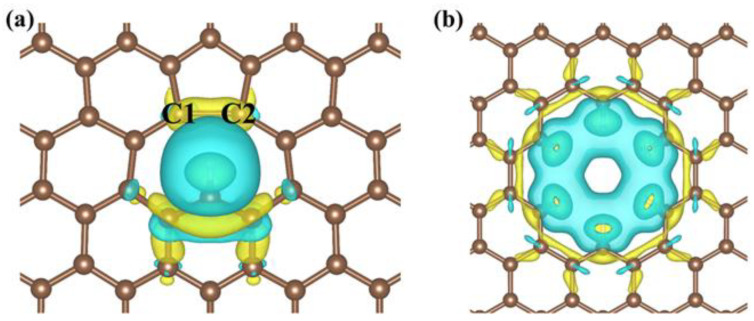
Differential charge density of (**a**) void-graphene and (**b**) hole-graphene catalysts. Yellow/blue represents charge accumulation/depletion, where the isosurfaces refer to isovalues of 1 × 10^−3^ and 2 × 10^−3^ *e*/bohr^3^ for (**a**) and (**b**), respectively.

As indicated by our calculations, a single-atom vacancy defect is able to promote the ECO_2_RR activity of graphene more significantly than a vacancy defect formed by multiple missing atoms. The intrinsic difference between these two kinds of vacancies is that the single-atom vacancy has a stronger interaction with the reactant CO_2_. The adsorption energies of CO_2_ on the surface of void-graphene, void-NPC, and void-NBPC catalysts are in the range of −1.48 to −0.64 eV. Nevertheless, the adsorption energies of CO_2_ on the catalysts with a multiatom vacancy defect (hole-graphene, hole-NPC, and hole-NBPC) are all higher than −0.20 eV and do not show advantages over pristine graphene. To rationalize these results, differential charge density calculations (Figure 6) were performed on the void-graphene and hole-graphene catalysts. The calculation results indicate that the void defect formed by a single missing atom and the hole defect formed by multiple missing atoms could cause distinct electronic charge redistribution in graphene. The hole defect results in a delocalized electron population around the defect, which forms an electron-deficient centre. In the case of the single-atom vacancy defect, the electron population accumulates on the side of the vacancy, which misses a carbon atom and forms a distorted five-member carbon ring (C1–C2, as shown in Figure 6a). A catalytic site is formed that favours the adsorption and activation of CO_2_. As mentioned in the results section, the electronic charge that is transferred from the catalyst to CO_2_ increases from 0.01 *e* in hole-graphene to 0.64 *e* in void-graphene, such that the CO_2_ molecule adsorbed on void-graphene converts to a bending configuration. In the cases of pristine graphene and hole-graphene, the adsorbed CO_2_ maintains a linear symmetrical conformation.

#### 3.2.2. Single-Atom Doping

Based on the adsorption energy of CO_2_ on various graphene catalysts, as listed in Table 1, the introduction of N into the graphene catalyst barely changes its ability to adsorb CO_2_. N doping in void-graphene and hole-graphene only reduces the adsorption energies of CO_2_ by 0.04–0.05 eV. However, pyridinic N doping considerably enhances the ability of the catalyst to adsorb atomic H. As discussed in the results section, pyridinic N doping generates a negatively charged spot on the surface of graphene. For each of the catalysts doped with pyridinic N, including void-NPC, hole-NPC, void-NBPC, hole-NBPC, and void-NBPC-O, the atomic H attaches to the pyridinic N and forms a N-H bond, which strongly polarizes H and facilitates the proton transfer process in the CO_2_→COOH reduction reaction step.

Unlike N-atom doping, single-boron-element doping does not result in structural defects but generates an electron-deficient centre in BPC (Figure S10), which is not able to promote the ability of the catalyst to adsorb CO_2_. Nevertheless, B doping does result in local polarization of the catalyst; thus, BPC has a stronger interaction with the polar reaction intermediate *COOH, which rationalizes the calculation results that the overpotential of BPC (1.27 eV) is lower than that of pristine graphene (2.20 eV). However, the improvement in the catalytic activity with single-B-atom doping is still very limited.

#### 3.2.3. Synergistic Effect between Doped Heteroatoms

As discussed above, single-B-atom doping only has a very slight effect on the catalytic activity of graphene. Although single-N-atom doping is able to provide better performance, it does not achieve satisfactory catalytic efficiency. Interestingly, after codoping the B atom with N, as shown in the results section, the catalytic activity from void-NPC to void-NBPC is prominently enhanced, indicating that doped N and B in graphene are able to work synergistically. By examining the calculation results, the most significant improvement in void-NBPC compared with void-NPC and NPC is that it effectively promotes the first reduction step of CO_2_ (CO_2_→*COOH), which can originate from either the activation of the reactant CO_2_ or stabilization of the intermediate *COOH.

As indicated by the calculated adsorption energies of CO_2_ on pristine graphene, BPC, and void-NPC, single-B-atom doping contributes little to the adsorption of CO_2_. The adsorption energy of CO_2_ on BPC is only −0.15 eV. The void defect resulting from N-doping could interact and activate CO_2_ such that the adsorption energy decreases to −0.68 eV on void-NPC. On void-NBPC, the adsorption energy of CO_2_ after N/B-codoping further decreases to −1.48 eV, which reflects the evident synergistic effect between B and N in the adsorption of CO_2_. 

**Figure 7:**
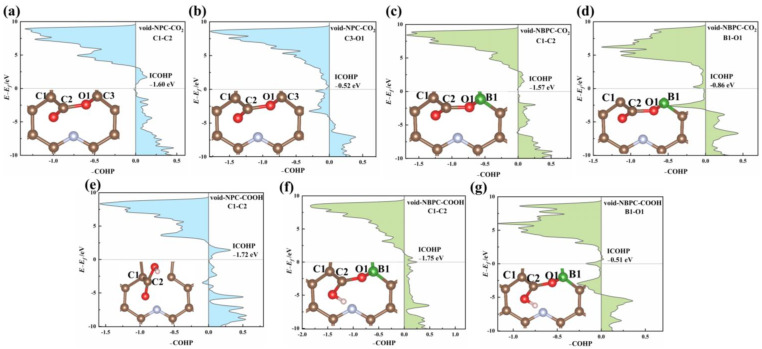
COHP of CO_2_ adsorbed on the surface of (**a**,**b**) void-NPC and (**c**,**d**) void-NBPC catalysts. The COHP of intermediate COOH adsorbed on the surface of (**e**) void-NPC and (**f**,**g**) void-NBPC catalysts. The brown, green, blue, red, and pink spheres represent carbon, boron, nitrogen, oxygen, and hydrogen atoms, respectively.

We further analysed the geometric structures of the CO_2_/catalyst complexes. On the surface of void-NPC, CO_2_ adopts a side-on conformation, which forms a C-C bond and a C-O bond with the catalyst (C1-C2 bond and C3-O1 bond, as shown in Figure 7a,b). In the complex with void-NBPC, the C-C bond between CO_2_ and the catalyst remains (C1-C2 bond in Figure 7c), while the C-O bond is replaced with a B-O bond (B1-O1 bond in Figure 7d). To obtain an in-depth understanding of the interaction between CO_2_ and the catalysts, the crystal orbital Hamilton population (-COHP) and the integral value of COHP below the Fermi level (ICOHP) analysis were performed [49,50,51]. The bonding and anti-bonding states are represented by positive and negative values in -COHP analysis, respectively. Accordingly, ICOHP is an index that reflects the interaction strength between the intermediates and the catalyst surface. The calculation results for ICOHP indicate that from the complex of CO_2_ with void-NPC to the complex with void-NBPC, the strength of the C-C bond between CO_2_ and the catalyst remains essentially unchanged (Figure 7a,c). However, the B-O bond formed between CO_2_ and void-NBPC is evidently stronger than the C-O bond between CO_2_ and void-NPC (Figure 7b,d) because the range of the antibonding states of the B-O bond between CO_2_ and void-NBPC is narrower than that of the C-O bond between CO_2_ and void-NPC. The stronger B-O bond between CO_2_ and void-NBPC should be due to the electron deficiency characteristic of B, which accommodates the electrons transferred from the C=O bond activated with the catalyst. Hence, the existence of B could further enhance the interaction between the catalyst and the reactant CO_2_ captured by the defect resulting from N-doping.

The *COOH intermediate generated from the first reduction step also shows a significantly stronger interaction with void-NBPC than with void-NPC. In its complex with void-NPC, *COOH only forms a C-C bond with the catalyst with a length of 1.53 Å (C1-C2 bond, as shown in Figure 7e). On the surface of void-NBPC, *COOH also uses its acyl O to form a B-O bond with the catalyst, for which the ICOHP is calculated to be −0.51 eV (B1-O1 bond as shown in Figure 7g). This B-O bond weakens the C=O bond in the *COOH intermediate but enhances the C-C bond between COOH and the catalyst such that it extremely shortens to 1.41 Å. As shown in Figure 7f, the antibonding states below the Fermi level of the C1-C2 bond disappear. The absolute value of ICOHP for the C1-C2 bond increases accordingly. The free energy change in the process CO_2_→*COOH on void-NPC is 0.94 eV, while that on void-NBPC is −0.37 eV, which also confirms the synergistic effect between the doped B and N in void-NBPC in its interaction with the intermediate *COOH.

#### 3.2.4. Doping Effects of the O Atom

Two models, BPC-O and void-NBPC-O, which are doped with O, were examined. BPC-O is the model that is based on BPC and further doped with two O atoms. Compared with BPC, BPC-O is able to adsorb the reactant H atom more strongly and does not show better performance in catalytic abilities.

**Figure 8:**
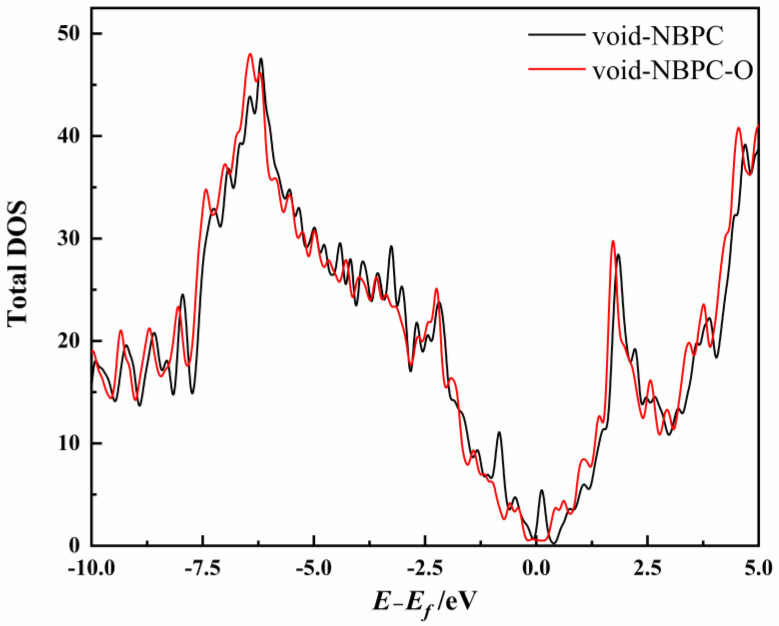
DOS of the void-NBPC and void-NBPC-O catalysts.

However, doping O in void-NBPC significantly alters the catalytic reaction process. Although void-NBPC-O does not show better performance in the adsorption of CO_2_ and in catalysing the conversion of CO_2_ to CO than void-NBPC, it remarkably reverses the process of CO desorption to be spontaneous. Comparing the structures of void-NBPC-O with void-NBPC, the doped O atoms reshape the void defect on the catalyst surface. As shown in Figure 1h, in void-NBPC, the absence of one C atom forms a five-membered C_4_B ring fused with a nine-membered C_8_N ring. The high tension in the five-membered C_4_B ring can easily be opened to react with the adsorbed CO_2_ catalytic reaction process, which accordingly promotes the activity of the catalyst. In its complex with the product CO, the carbon terminal of CO exactly fills the void defect such that the complex structure is close to quasi-perfect graphene, resulting in a very high cost to remove CO from the catalyst surface and inevitably leading to poisoning of the catalyst. In void-NBPC-O, the doped O atoms form a BCO_2_ species with the doped B atom. Although there also exists a void defect in void-NBPC-O, the high-tension five-membered ring no longer exists due to multiple heteroatom doping. Hence, by slightly compromising its catalytic activity, void-NBPC-O is circumventing CO poisoning. We further analysed the electronic structure of void-NBPC and void-NBPC-O. The DOS analysis indicates that in void-NBPC, there is an empty energy level at *E* − *E_f_* = 0.12 eV (Figure 8), which is able to accept the electrons transferred from CO to form back-feed bonding. This empty energy level could originate from either the doped electron-deficient B atom or the void defect in the catalyst surface. However, in void-NBPC-O, this virtual energy level vanishes because the excess electrons brought by the doped oxygen fill it, which in turn weakens the interaction between the catalyst and CO and facilitates the release of the product.

## 4. Conclusions

In this study, DFT calculations were used to investigate the underlying ECO_2_RR mechanisms on a series of graphene-based catalysts. The catalytic reaction follows a common three-step process: CO_2_→*COOH→*CO→CO. In general, the potential determining step in the reaction lies in the first reduction reaction. In the case of N/B-codoped graphene with a single-atom vacancy defect, the catalyst is able to effectively promote the two reduction steps to convert CO_2_ into CO but has an excessively strong interaction with the product CO. Thus, the potential determining step in the complete reaction is the desorption of CO, which is extremely high and inevitably leads to poisoning of the catalyst. Our calculation indicates that N/B/O-codoped graphene with a single-atom vacancy has the best catalytic performance among all catalysts studied. At the equilibrium potential of *U_eq_* = −0.25 V, the required overpotential is only 0.64 eV. Based on the obtained catalytic reaction mechanisms, the roles of the defects and the doped heteroatoms, as well as the synergistic effect between them, are discussed. The detailed analysis reveals that the single-atom vacancy defect is able to enhance the adsorption and activation of CO_2_. Single-B-atom-doping barely changes the activity of the catalyst. Pyridinic N-doping promotes the binding of atomic H. Due to the electron-deficient characteristic of the B atom, codoping B with N in graphene could gain extra binding affinity to the reactant CO_2_ and the reaction intermediate *COOH by forming a B-O bond between the catalyst and the reaction species. Therefore, on the surface of catalyst void-NBPC, conversion from CO_2_ into *COOH intermediate is promoted. All catalysts are capable of spontaneously converting *COOH to *CO. Further introduction of O atoms into the N/B-codoped catalyst is able to reshape the geometry structure and electronic structures of the catalyst surface such that the adsorption of the product CO on the catalyst surface is suppressed, which effectively balances the activity of the catalyst and excessive interaction with the product. Hence, void-NBPC-O reaches the highest performance among all the catalysts studied in this work. Our work revealed how the doped heteroatoms and defects regulate the catalytic activity of graphene; based on our results, a strategy to create new catalysts with higher efficiency could be developed.

## Figures and Tables

**Table 1:** Adsorption energies (*E_ad_*) of CO_2_, CO, and H atoms on the surface of the 10 catalyst models in the solvation environment. All values are in eV.

	CO_2_	CO	H
graphene	−0.15	−0.13	1.31
void-graphene	−0.64	−3.00	−0.34
hole-graphene	−0.14	−0.13	0.26
BPC	−0.15	−0.14	0.56
BPC-O	−0.17	−0.14	−0.46
void-NPC	−0.68	−0.14	−0.75
hole-NPC	−0.19	−0.15	−0.42
void-NBPC	−1.48	−2.16	−1.00
hole-NBPC	−0.19	−0.15	−0.78
void-NBPC-O	−0.19	−0.15	−0.59

**Table 2:** Free energy data and overpotential of the 10 catalyst models for CO_2_ reduction in the solvation environment. An asterisk represents a free adsorption site. All values are in eV.

	CO_2_→*COOH	*COOH→*CO	*CO→CO	Overpotential
graphene	2.45	−1.64	−0.31	2.20
void-graphene	0.97	−2.76	2.29	0.72
hole-graphene	1.30	−0.45	−0.34	1.05
BPC	1.52	−0.76	−0.26	1.27
BPC-O	1.53	−0.72	−0.31	1.28
void-NPC	0.94	−0.18	−0.26	0.69
hole-NPC	1.30	−0.59	−0.21	1.05
void-NBPC	−0.37	−0.64	1.51	-
hole-NBPC	1.24	−0.52	−0.21	0.99
void-NBPC-O	0.89	−0.12	−0.27	0.64

## Data Availability

Data available on request.

## Supplementary Materials

The following supporting information can be downloaded at: https://www.mdpi.com/article/10.3390/nano13152273/s1, Figure S1: The constructed models of void-NBPC and hole-NBPC; Figures S2–S4: Optimized adsorption configurations of intermediates on the graphene, void-graphene and hole-graphene surface, respectively; Figure S5: ELF results for CO_2_ adsorbed on void-NPC and pristine graphene surface; Figures S6–S9: Optimized adsorption configurations of intermediates on the hole-NPC, BPC, BPC-O and hole-NBPC surface, respectively; Figure S10: Differential charge density of BPC catalyst.
