# Supplementary material for: Theoretical Study of the Defects and Doping in Tuning the Electrocatalytic Activity of Graphene for CO2 Reduction
Source: Nanomaterials (Basel). 2023 Aug 7;13(15):2273. doi: 10.3390/nano13152273 (PMC10421040; doi:10.3390/nano13152273)
Supplement: Supplementary file 1 [file nanomaterials-13-02273-s001.zip › nanomaterials-2531734-supplementary.pdf]

Theoretical Study of the Defects and Doping in Tuning the Electrocatalytic Activity of Graphene for CO_2 Reduction

Xiao Su[1], Fanqi Meng[1], Xiang Li[1], Yueying Liu[1], Hongwei Tan[1,*], Guangju Chen[1,*]

1. Key Laboratory of Theoretical and Computational Photochemistry, Ministry of Education, College of Chemistry, Beijing Normal University, Beijing 100875, China

* Authors to whom correspondence should be addressed.

E-mail address: hongwei.tan@bnu.edu.cn

gjchen@bnu.edu.cn

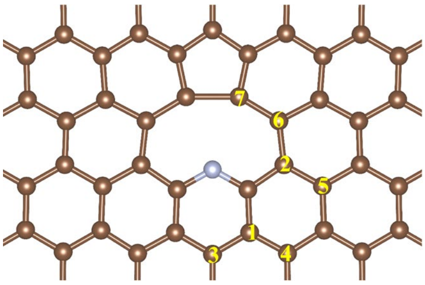

	E_{total}	E_{gap}
1	2.15	1.21
2	1.53	0.92
3	2.10	1.22
4	2.00	1.28
5	1.65	1.17
6	0.81	1.19
7	0	0.94

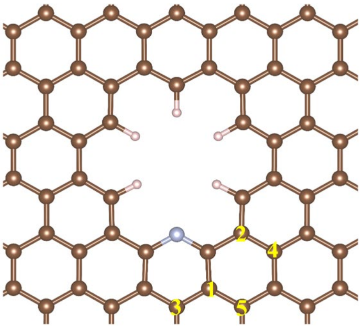

	E_{total}	E_{gap}
1	0.15	1.28
2	0.06	1.22
3	0.14	1.44
4	0.04	1.23
5	0	1.18

Figure S1. The constructed models of (a) void-NBPC and (b) hole-NBPC. The yellow serial marks represent the positions of the doped B atoms. E_{total} is the relative energy, the No. 7 of void-NBPC and No. 5 of hole-NBPC structures with the lowest energies were selected as the benchmark. The brown, blue, green and pink spheres represent carbon, nitrogen, boron and hydrogen atoms, respectively.

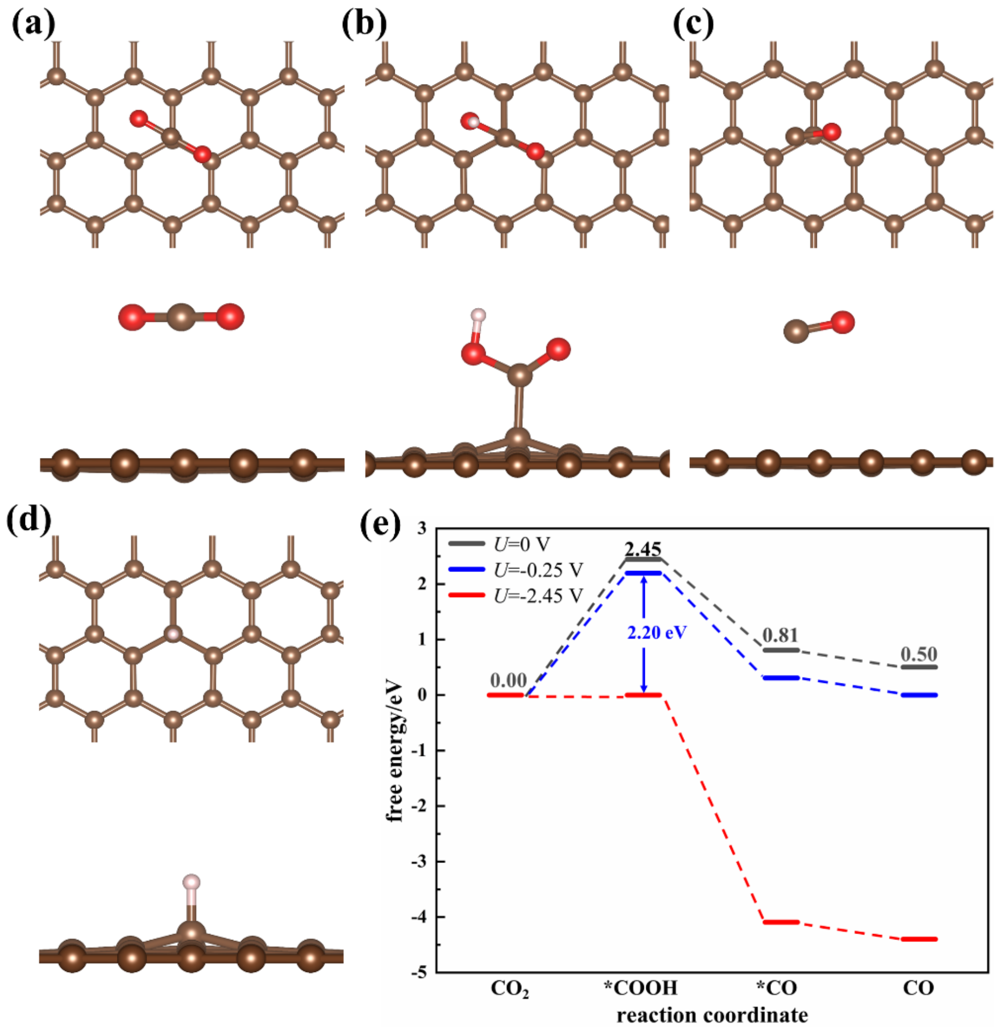

Figure S2. Optimized adsorption configurations of (a) CO_2, (b) COOH, (c) CO and (d) H on the pristine graphene surface. (e) Free energy profile of CO_2 reduction on the pristine graphene surface in the solvation environment at different applied potentials. The brown, red and pink spheres represent carbon, oxygen and hydrogen atoms, respectively.

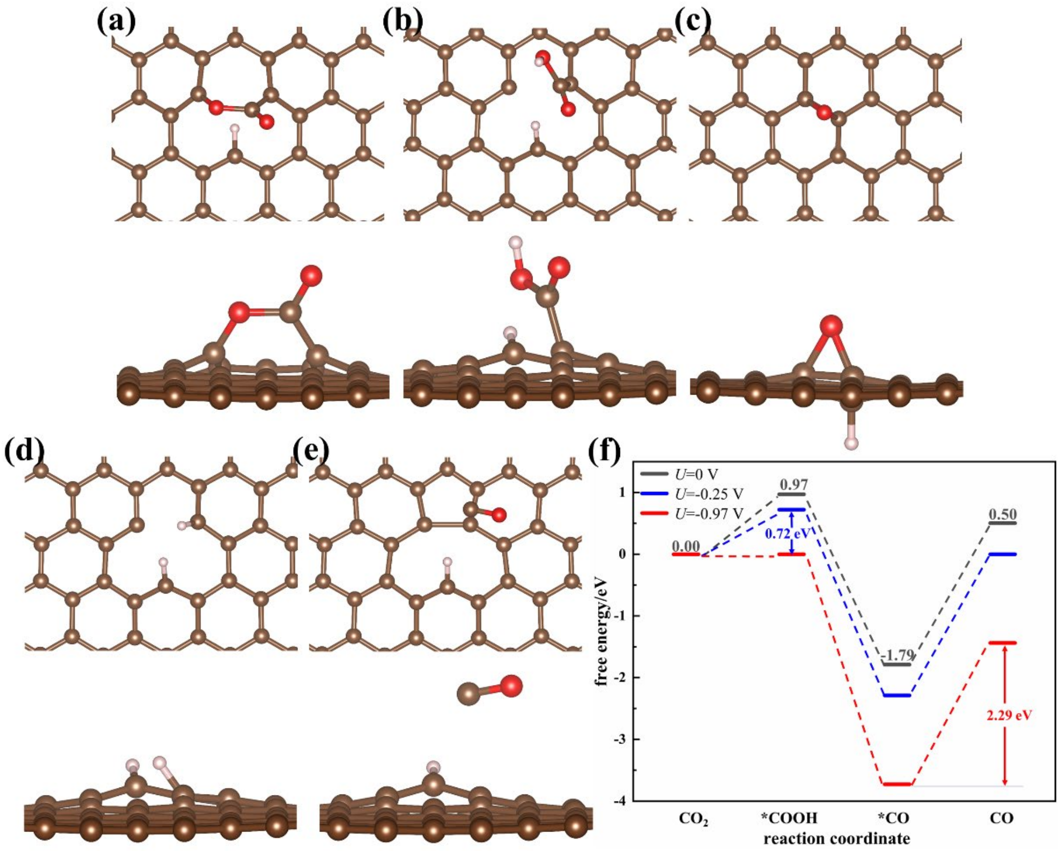

Figure S3. Optimized adsorption configurations of (a) CO_2, (b) COOH, (c) adsorption state CO, (d) H and (e) dissociative state CO on the void-graphene surface. (f) Free energy profile of CO_2 reduction on the void-graphene surface in the solvation environment. The brown, red and pink spheres represent carbon, oxygen and hydrogen atoms, respectively.

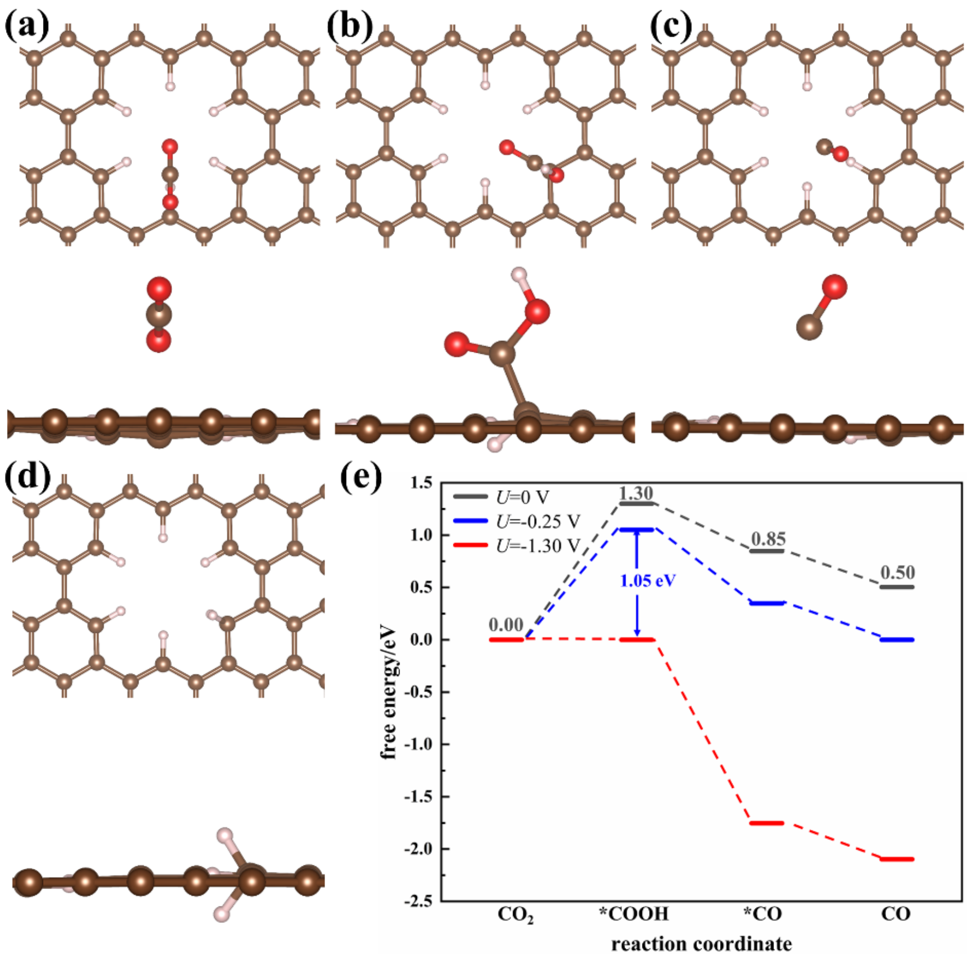

Figure S4. Optimized adsorption configurations of (a) CO_2, (b) COOH, (c) CO and (d) H on the hole-graphene surface. (e) Free energy profile of CO_2 reduction on the hole-graphene surface in the solvation environment at different applied potentials. The brown, red and pink spheres represent carbon, oxygen and hydrogen atoms, respectively.

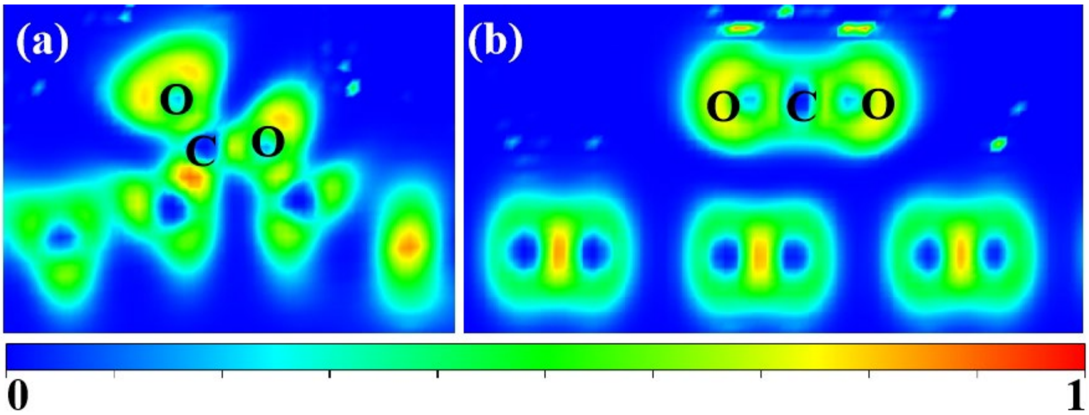

Figure S5. ELF results for CO_2 adsorbed on (a) void-NPC and (b) pristine graphene surface.

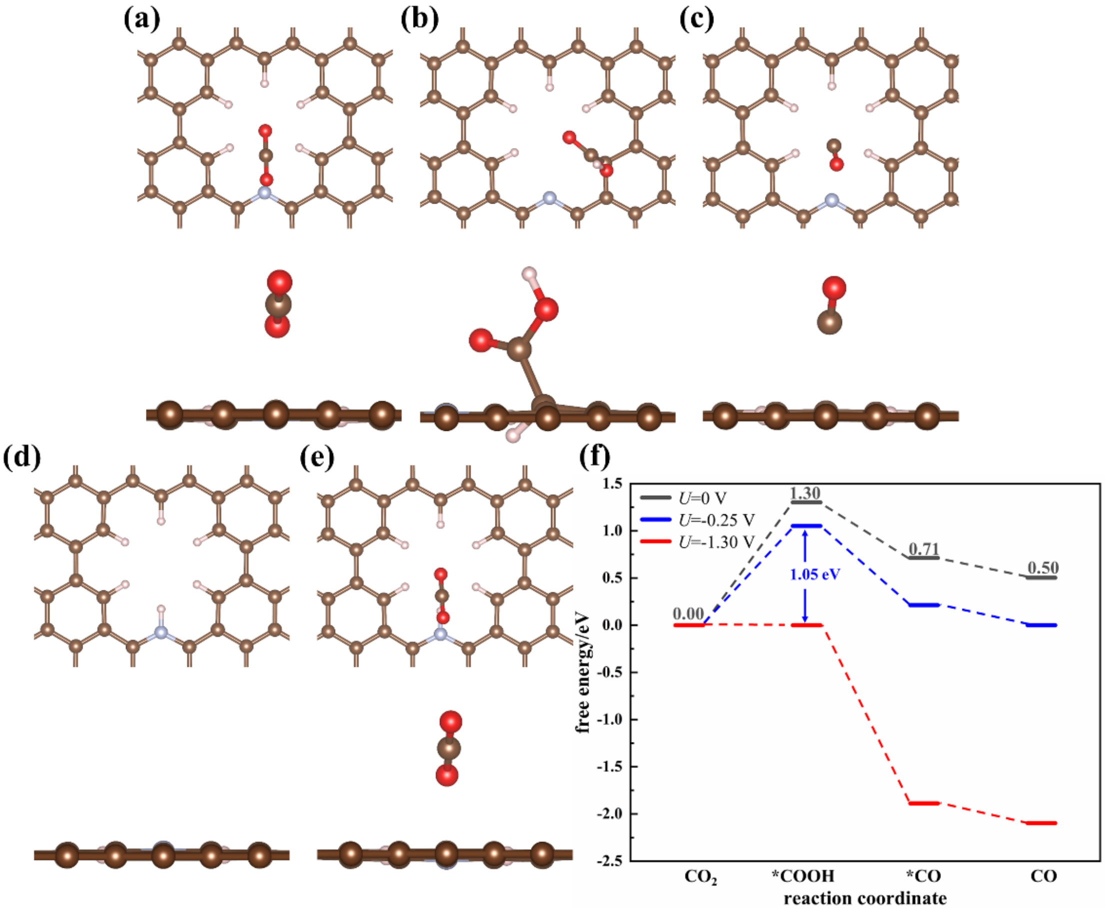

Figure S6. Optimized adsorption configurations of (a) CO_2, (b) COOH, (c) CO, (d) H and (e) CO_2/H on the hole-NPC surface. (f) Free energy profile of CO_2 reduction on the hole-NPC surface in the solvation environment at different applied potentials. The brown, blue, red and pink spheres represent carbon, nitrogen, oxygen and hydrogen atoms, respectively.

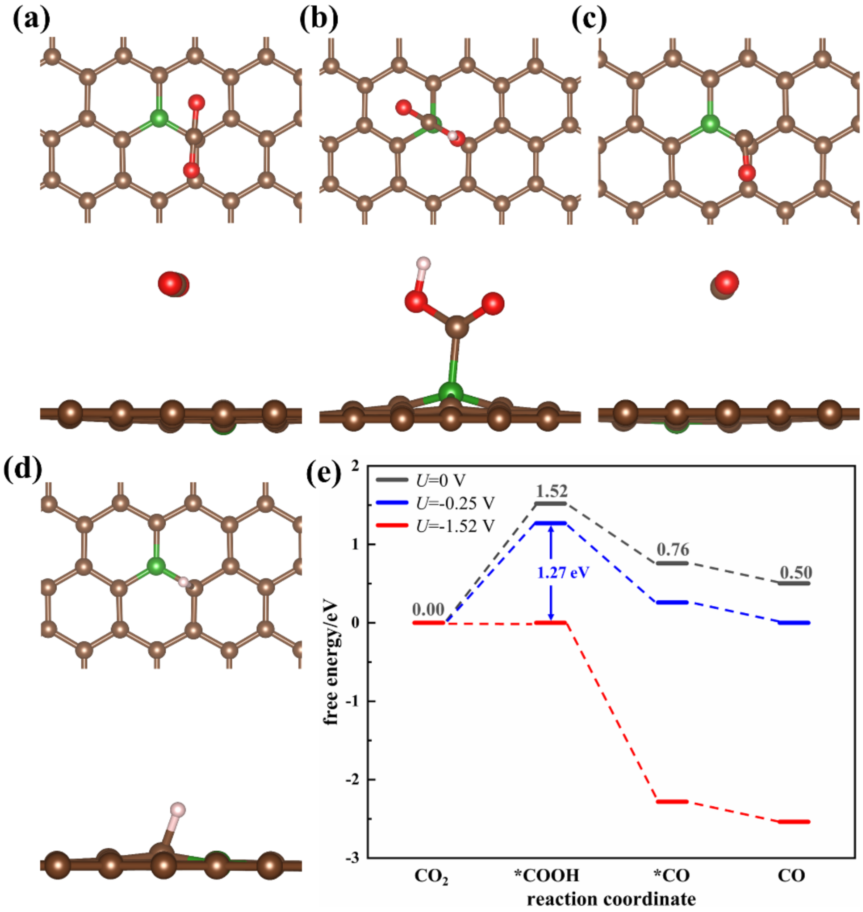

Figure S7. Optimized adsorption configurations of (a) CO, (b) COOH, (c) CO and (d) H on the BPC surface. (e) Free energy profile of CO_2 reduction on the BPC surface in the solvation environment at different applied potentials. The brown, green, red and pink spheres represent carbon, boron, oxygen and hydrogen atoms, respectively.

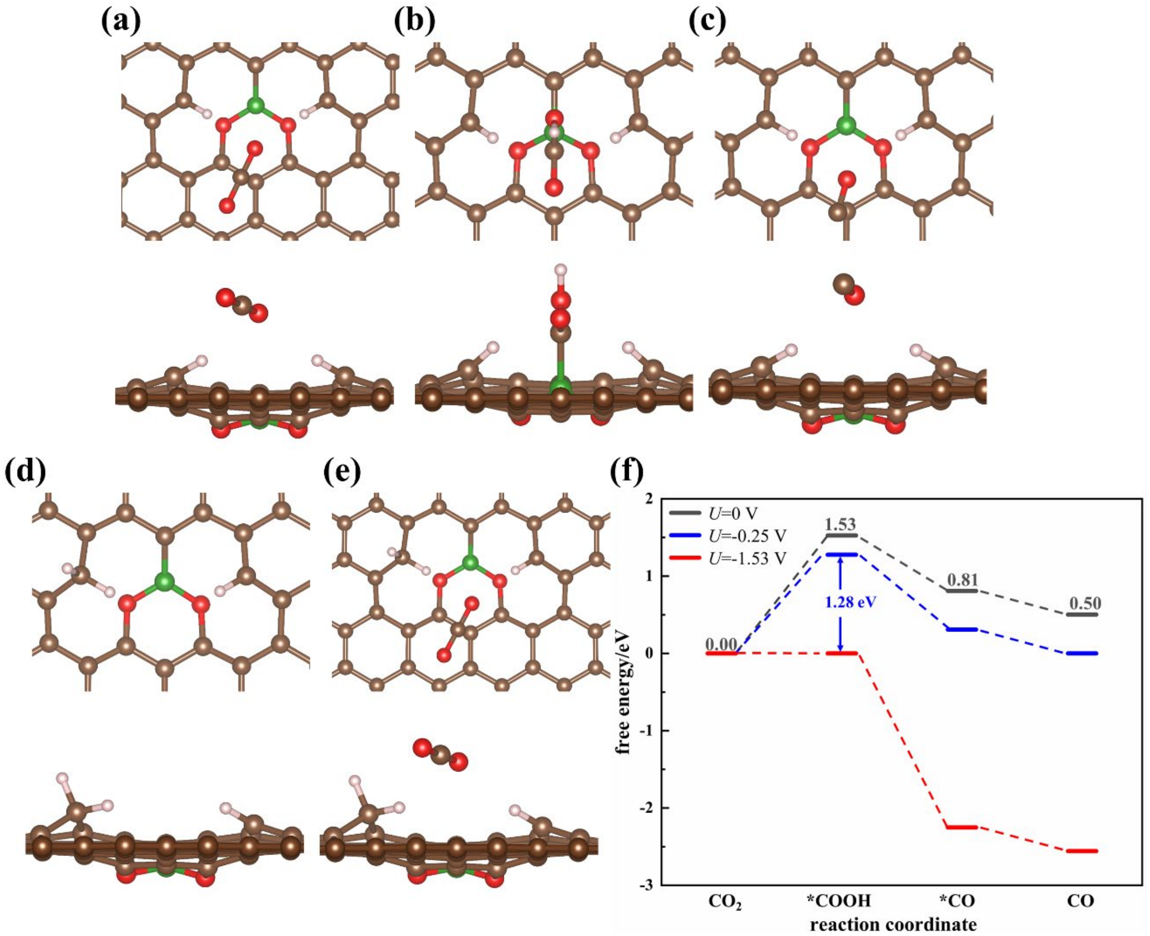

Figure S8. Optimized adsorption configurations of (a) CO_2, (b) COOH, (c) CO, (d) H and (e) CO_2/H on the BPC-O surface. (f) Free energy profile of CO_2 reduction on the BPC-O surface in the solvation environment at different applied potentials. The brown, green, red and pink spheres represent carbon, boron, oxygen and hydrogen atoms, respectively.

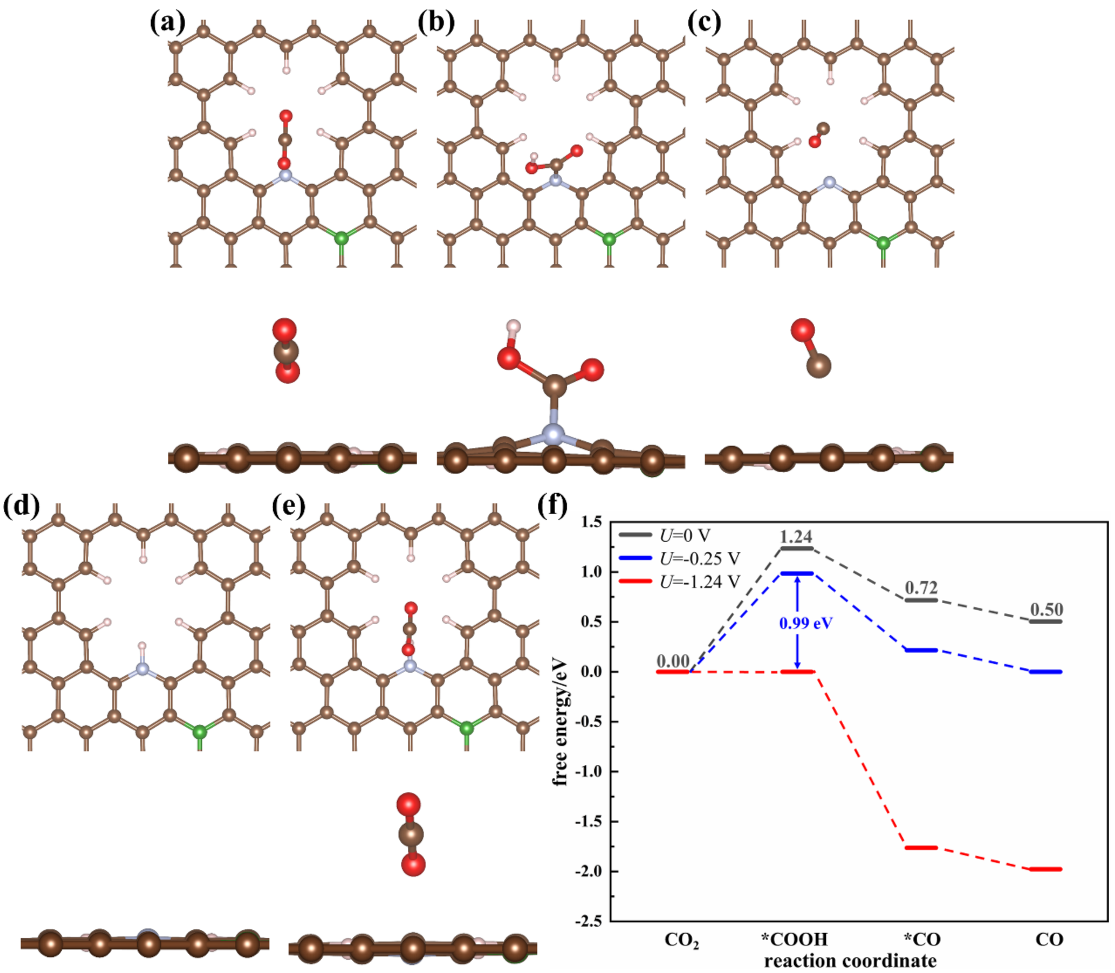

Figure S9. Optimized adsorption configurations of (a) CO_2, (b) COOH, (c) CO, (d) H and (e) CO_2/H on the hole-NBPC surface. (f) Free energy profile of CO_2 reduction on the hole-NBPC surface in the solvation environment at different applied potentials. The brown, green, red and pink spheres represent carbon, boron, oxygen and hydrogen atoms, respectively.

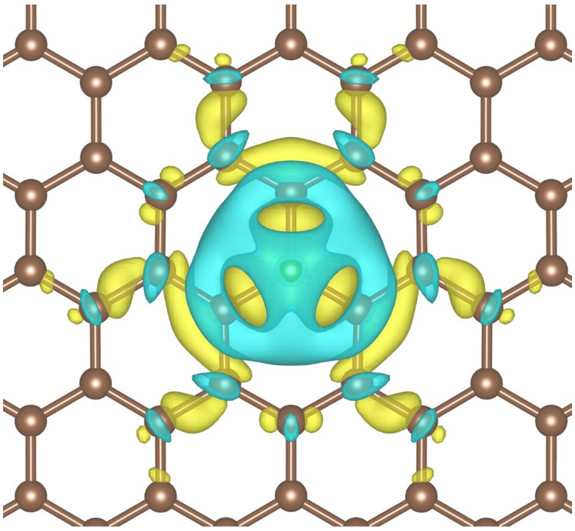

Figure S10. Differential charge density of BPC catalyst. Yellow/blue represents charge accumulation/depletion, where the isosurfaces refer to isovalues of 2×10^{-3} *e*/$bohr^3$.
